# Multi-Stress Induction of Astaxanthin Accumulation in *Haematococcus lacustris:* A Taguchi-Designed Experimental Study

**DOI:** 10.3390/md24100350

**Published:** 2026-10-04

**Authors:** Christina Samara, Despoina Samolada, Evangelia Matzarli, Georgia Papapanagiotou, Christos Chatzidoukas

**Affiliations:** Laboratory of Biochemical and Biotechnological Processes (LB^2^P), Department of Chemical Engineering, Aristotle University of Thessaloniki (AUTH), 54124 Thessaloniki, Greece; cdsamara@auth.gr (C.S.); dsamolad@auth.gr (D.S.); matzarlie@auth.gr (E.M.); papapana@auth.gr (G.P.)

**Keywords:** inductive stage cultivation, temperature, CO_2_, salinity, intermittent gas supply

## Abstract

Multi-stress induction represents a promising strategy for enhancing astaxanthin accumulation in *Haematococcus lacustris*; however, productivity optimization requires balancing cellular enrichment with biomass formation. This study investigates the main effects of three critical process variables, namely temperature, CO_2_ supplementation, and salinity, on the induction of astaxanthin in autotrophic cultivation. A Taguchi L9 orthogonal-array design elucidated how the combined variation in the above factors, across three levels each, affected biomass formation, astaxanthin biosynthesis and biochemical composition under nitrogen and phosphorus deprivation and continuous illumination. Duplicate cultures were incubated for 12 days in 500 mL bottles sparged daily with CO_2_–air mixtures until target outlet concentrations were reached and sealed afterwards. The response-specific main-effect analysis indicated that CO_2_ governed biomass and volumetric astaxanthin production, whereas salinity dominated in astaxanthin and lipid content regulation. Maximum values attained for the set engineering objectives included 4.23 wt.% for astaxanthin content and 2.49 g/L for biomass concentration. Model R^2^ ranged from 87.97% to 97.57%, while predicted R^2^ exceeded 80% for all responses except protein content. An independent validation experiment at the model-predicted optimum (28 °C, 9% CO_2_, and 6 g/L NaCl) yielded 101.0 mg/L astaxanthin, supporting the model’s utility within the investigated factor levels.

## 1. Introduction

The growing demand for sustainable, naturally derived ingredients has positioned microalgae at the forefront of blue biotechnology, particularly as sources of high-value bioactive compounds [1]. Among these compounds, astaxanthin (3,3′-dihydroxy-*β*,*β*-carotene-4,4′-dione) is a secondary ketocarotenoid valued for its potent antioxidant and anti-inflammatory activities and its applications in aquaculture feeds, foods, nutraceuticals, and cosmetics [2,3]. In cosmetic and nutraceutical applications, astaxanthin has been investigated for its capacity to mitigate oxidative and inflammatory damage, including UV-associated skin damage, while human studies have reported improvements in skin moisture, elasticity, texture, and wrinkle appearance [3,4]. These functional properties and expanding applications have intensified interest in efficient natural astaxanthin production.

Among natural producers, the freshwater microalga *Haematococcus lacustris* (synonym *Haematococcus pluvialis*) [5] is recognized as the principal commercial source and can accumulate astaxanthin at approximately 3–5% of dry biomass under inductive conditions [6,7]. Most of the pigment is deposited as mono- and di-esters within lipid droplets, which contribute to its intracellular stabilization and closely associate astaxanthin formation with lipid metabolism [8]. Nevertheless, the relatively slow growth of *H. lacustris*, together with the requirement for growth-limiting conditions to induce secondary carotenogenesis, makes volumetric astaxanthin production highly dependent on cultivation strategy [9,10]. Efficient production must therefore balance cellular astaxanthin enrichment with the preservation of sufficiently high culture density, since the condition producing the highest astaxanthin content does not necessarily maximize pigment volumetric concentration [11].

Commercial production consequently relies predominantly on a two-stage cultivation strategy comprising a vegetative (green) stage for cell proliferation and an inductive (red) stage for astaxanthin induction [6,7]. During the vegetative stage, nutrient-replete conditions and moderate illumination support cell proliferation and biomass accumulation. In the subsequent inductive stage, environmental and nutritional stresses induce the transition from motile green cells to non-motile, thick-walled aplanospores, accompanied by extensive remodeling of the photosynthetic apparatus and progressive astaxanthin accumulation [7,11]. Increased oxidative pressure is central to this transition. Astaxanthin synthesis and deposition protect cells against photooxidative damage, while pigment formation is synchronous with fatty-acid synthesis and triacylglycerol accumulation [8,12,13]. However, the same stresses that activate carotenogenesis can suppress cell division and impair photosynthetic performance [14], and excessive or prolonged culture exposure reduces cell viability [15]. Biomass concentration, astaxanthin content, and volumetric astaxanthin concentration must therefore be regarded as distinct and potentially competing engineering objectives.

Nutrient availability and illumination provide the principal background for the induction stage. Nitrogen deprivation promotes the degradation of nitrogen-rich proteins and photosynthetic components and redirects cellular carbon toward storage carbohydrates, fatty acids, and secondary carotenoids [16]. Phosphorus availability and illumination intensity and duration further influence the balance between biomass retention and astaxanthin accumulation [17,18]. Increased or prolonged illumination intensifies excitation pressure and reactive oxygen species generation, thereby promoting carotenogenesis, whereas excessive exposure can cause photoinhibition and cellular damage [13,18]. Temperature and salinity impose additional thermal, osmotic, and oxidative pressures. Astaxanthin productivity under nitrogen starvation, for example, was higher at 27 °C than at 20 °C, although a further temperature rise strongly restricted cell accumulation [19]. Similarly, low-to-moderate NaCl supplementation can stimulate astaxanthin and lipid accumulation and, under suitable two-stage conditions, improve volumetric pigment production, whereas more severe saline stress progressively impairs photosynthetic performance and biomass accumulation [20,21]. Each factor therefore has a favorable operating range beyond which additional stress becomes counterproductive and restraining.

CO_2_ on the other hand differs from these factors because it primarily supplies inorganic carbon rather than acting as a stressor per se. Moderate CO_2_ culture enrichment can support photosynthesis, biomass growth, and volumetric astaxanthin accumulation, whereas excessive supply may acidify the medium, disrupt photochemical performance, or become inhibitory depending on gas concentration, exposure regime, and culture-buffering capacity [22,23]. Continuous CO_2_ sparging is often used to maintain carbon availability; however, inefficient gas–liquid transfer or supply exceeding biological demand can imply wasteful gas consumption and a concomitant increase in the energy and cost associated with carbon delivery [24]. The gas concentration and delivery strategy should therefore be considered separately when evaluating the effects of inorganic-carbon supply.

Combined and sequential induction strategies have coupled nitrogen starvation with high irradiance [18], elevated CO_2_ with increased illumination [23], NaCl with culture age and sodium acetate [25], and sequential nutrient and light stresses [26]. Plant-growth regulators have also been combined with nutrient deprivation, salinity, and high irradiance to promote astaxanthin and lipid co-accumulation [27]. Furthermore, response surface methodology has also been implemented to distinguish conditions favoring biomass production from those favoring carotenogenesis [28], or to optimize the combination of light intensity, sodium acetate, and salicylic acid for astaxanthin accumulation [29]. The research gap is therefore not an absence of multi-stress optimization studies, but the heterogeneity of the investigated factors, factor levels, exposure strategies, and performance criteria. In particular, the response-specific effects of temperature, gaseous CO_2_ supply, and salinity across multiple levels remain insufficiently resolved under a common inductive background of complete nitrogen and phosphorus deprivation and continuous illumination. These variables have rarely been evaluated simultaneously but typically are tested alongside other factors. Temperature is often studied with light or nitrogen starvation [19,30,31,32], salinity is usually paired with light or other stressors [25,33,34], and CO_2_ is examined with pH or with the feeding strategy [35,36] rather than with a salinity–temperature combination.

Against this background, the present study applied a Taguchi L9 orthogonal-array design as an economical main-effects screening approach to elucidate and quantify the effects of temperature (22, 25, and 28 °C), supplied gaseous CO_2_ concentration (3, 6, and 9% *v*/*v*), and salinity (0, 3, and 6 g/L NaCl) during the inductive stage of *H. lacustris*. All cultures were evaluated under complete nitrogen and phosphorus deprivation and continuous illumination at 200 μmol/m^2^/s, applying an established inductive background against which the investigated factors could be compared. Building on the previous application of Taguchi design to vegetative stage optimization [37], the present study jointly evaluates biomass formation, astaxanthin content and volumetric concentration, together with carbohydrate, protein, and lipid contents, while quantifying the relative contribution of each factor and developing response-specific fitted models. The principal contribution therefore lies in: (i) distinguishing factor levels that favor cellular astaxanthin enrichment from those supporting biomass-associated volumetric production, (ii) demonstrating that a single cultivation condition does not optimize all inductive stage outcomes, and (iii) implementing a response-specific optimization strategy supported by the experimental design framework. In the present experimental system, cultivation proceeded in a totally sealed setup where the desired CO_2_–air atmosphere in the culture headspace was renewed only once daily rather than being continuously supplemented. Although not directly investigated, this strategy provides a basis for future evaluation of intermittent CO_2_ delivery as a potential alternative to continuous dosing during astaxanthin induction.

## 2. Results and Discussion

### 2.1. Biomass Formation and Astaxanthin Accumulation

**Figure 1:**
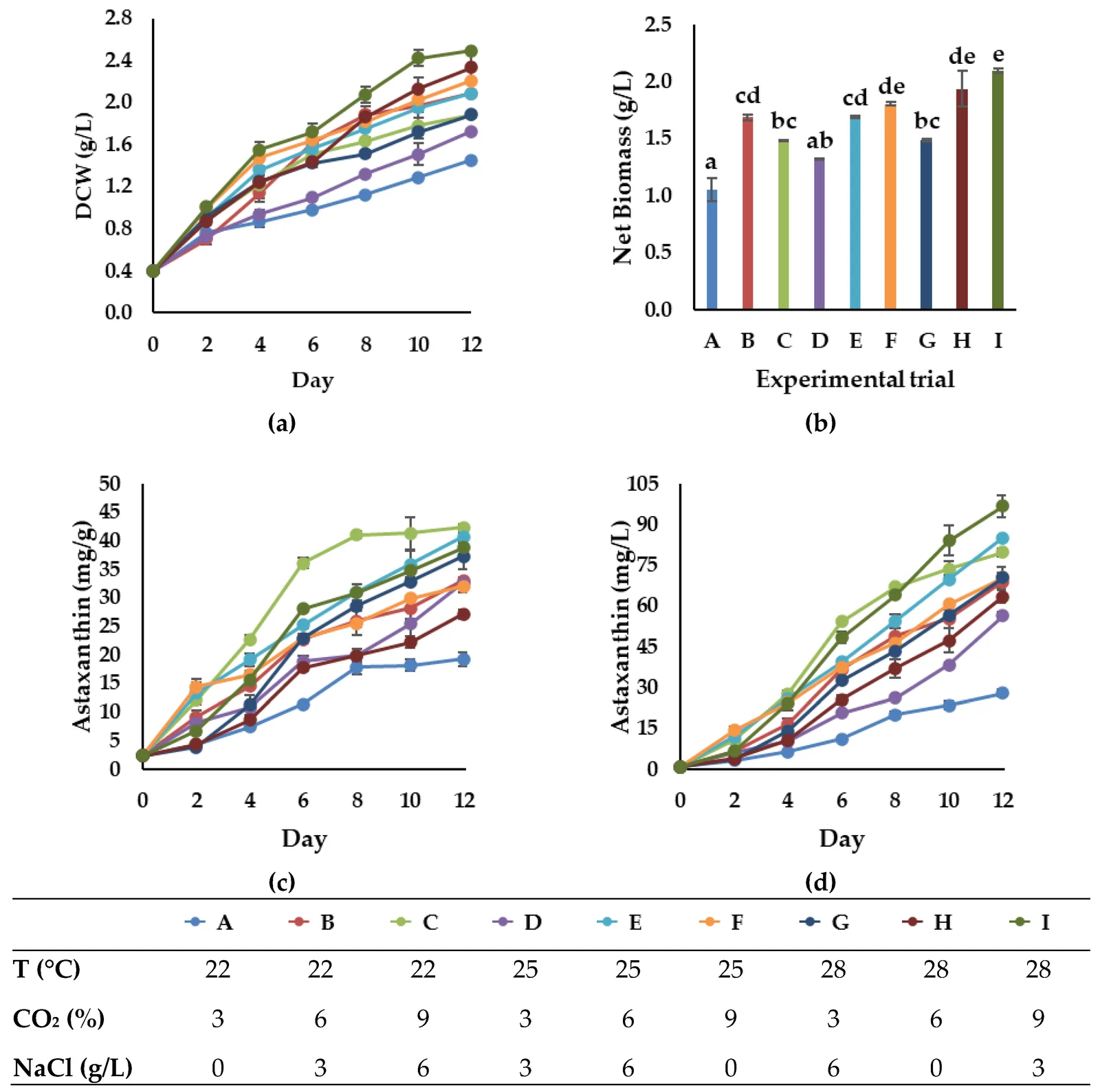
Production of (**a**) total biomass; (**b**) net biomass; (**c**) astaxanthin content and (**d**) astaxanthin concentration. Error bars represent the standard deviation of the two biological replicates. Responses not sharing a letter are statistically different as detected by Tukey’s pairwise comparison at *p* < 0.05. The embedded table summarizes the operational parameters of the nine experimental trials.

**Figure 2:**
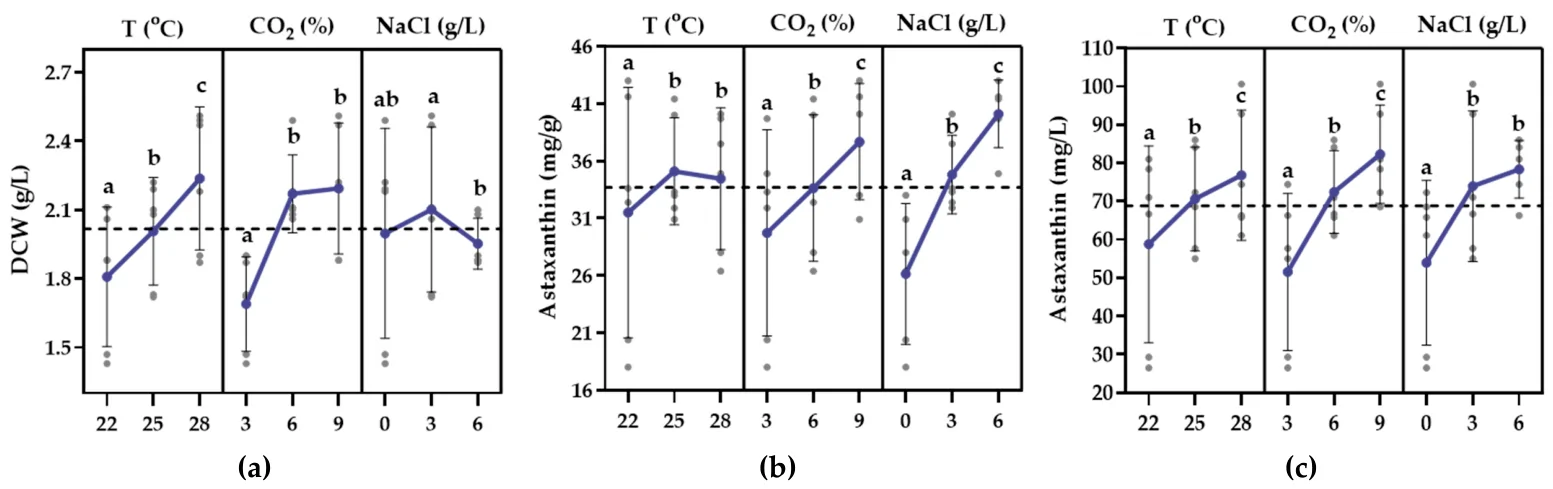
Main effects plots for (**a**) biomass; (**b**) astaxanthin content and (**c**) astaxanthin concentration. Gray dots represent the real values of the independent biological cultures, while purple dots represent the mean value of the 6 cultures sharing the same level. Error bars represent the 95% Confidence Interval (CI). The dashed line represents the total mean of the 18 independent biological cultures. Levels of each factor that do not share a letter are statistically different as detected by Tukey’s pairwise comparisons at *p* < 0.05.

Across the nine experimental cultures, dry cell weight (DCW) increased throughout the 12-day induction stage, rising from a common initial concentration of 0.40 g/L to final concentrations of 1.45–2.49 g/L (Figure 1a), demonstrating significant net biomass formation in nitrogen- and phosphorus-depleted medium under continuous illumination and daily headspace renewal with a CO_2_-enriched atmosphere. The achieved biomass yields confirm that the absence of nutrients and air supply did not compromise cell viability or culture survival. Culture I (28 °C, 9% CO_2_, and 3 g/L NaCl) reached the highest final DCW (2.49 g/L; 6.2-fold the initial concentration), followed by Cultures H and F (2.33 and 2.21 g/L, respectively). Cultures A, D, and G, all assigned the 3% CO_2_ level, had the lowest DCW within their respective temperature groups (1.45, 1.73, and 1.89 g/L, respectively), indicating that the 3% CO_2_ headspace was associated with the lowest biomass response and was insufficient to satisfy the cellular metabolic demands required to sustain rapid growth. These observed trends in DCW across treatments were directly reflected in the corresponding main effects plots, which visualize and quantify the influence of each individual factor on biomass production. As illustrated in Figure 2a, elevated temperatures favored biomass production, as higher temperature increases the size of *H. lacustris* cells resulting in overall higher total weight [19]. While CO_2_ levels in the culture headspace of 6% and 9% *v*/*v* yielded comparable biomass production, both resulted in significantly higher DCW than the 3% one. According to Li et al. [38], cultivation under high CO_2_ supply was associated with upregulation of carbon-fixation genes in *H. lacustris*, supporting biomass formation and the supply of precursors for astaxanthin biosynthesis. Notably, increasing salinity up to 3 g/L resulted in a marginal biomass increase; however, further elevation to 6 g/L caused a slight reduction in overall yield. This trend indicates that while moderate salinity slightly favors growth, higher salt concentrations suppress photosynthesis and restrict biomass production [20].

Cellular astaxanthin content increased from 2.3 mg/g in the inoculum to 19.2–42.3 mg/g on day 12 (Figure 1c). Culture C (22 °C, 9% CO_2_, and 6 g/L NaCl) exhibited the fastest enrichment, reaching 41.1 mg/g by day 8 and the maximum 42.3 mg/g (4.23% wt.) at a final DCW of 1.88 g/L. Under these conditions of high salinity, high carbon availability and low temperature, the majority of the supplied carbon was redirected into astaxanthin rather than biomass production. The stimulatory effect of salinity on astaxanthin accumulation is consistent with previous reports attributing this response to the upregulation of key carotenogenic genes [39]. Possibly, under carbon insufficiency, salinity-induced impairment of photosynthetic activity could restrict the synthesis of protective compounds (antioxidant shield) including astaxanthin and storage lipids, thereby limiting stress tolerance and pigment accumulation [20,40]. Cultures E and I (assigned with medium and high levels of all three factors) also approached 4% wt. (40.7 and 38.8 mg/g), producing more biomass than Culture C. The high astaxanthin and biomass yields of these cultures indicate that strong cellular enrichment was not necessarily associated with restricted biomass formation, but when inorganic carbon is provided at medium-to-high concentrations, it may preserve cell viability under salinity-induced osmotic stress long enough for carbon to be redirected into astaxanthin, with temperature determining how rapidly this redirection occurs. Thus, the potential destructive effect of high salinity on growth seems to be essentially mitigated by the enhanced carbon availability and its accelerated fixation under high temperature. Overall, while salinity and elevated carbon availability drive astaxanthin accumulation, higher temperatures are critical for enabling the concurrent production of both pigment and biomass. These observations about carbon availability and salinity are also reflected in the corresponding main effects plots for astaxanthin accumulation (Figure 2b), where both factors demonstrated a positive, dose-dependent relationship with astaxanthin content, leading to increments of up to 25 and 50% compared with the corresponding lowest tested levels. Although the lowest tested temperature resulted in a slightly reduced astaxanthin content, the differences compared with 25 and 28 °C remained limited, indicating that temperature within the investigated range played a secondary role in pigment enrichment.

**Figure A1:**
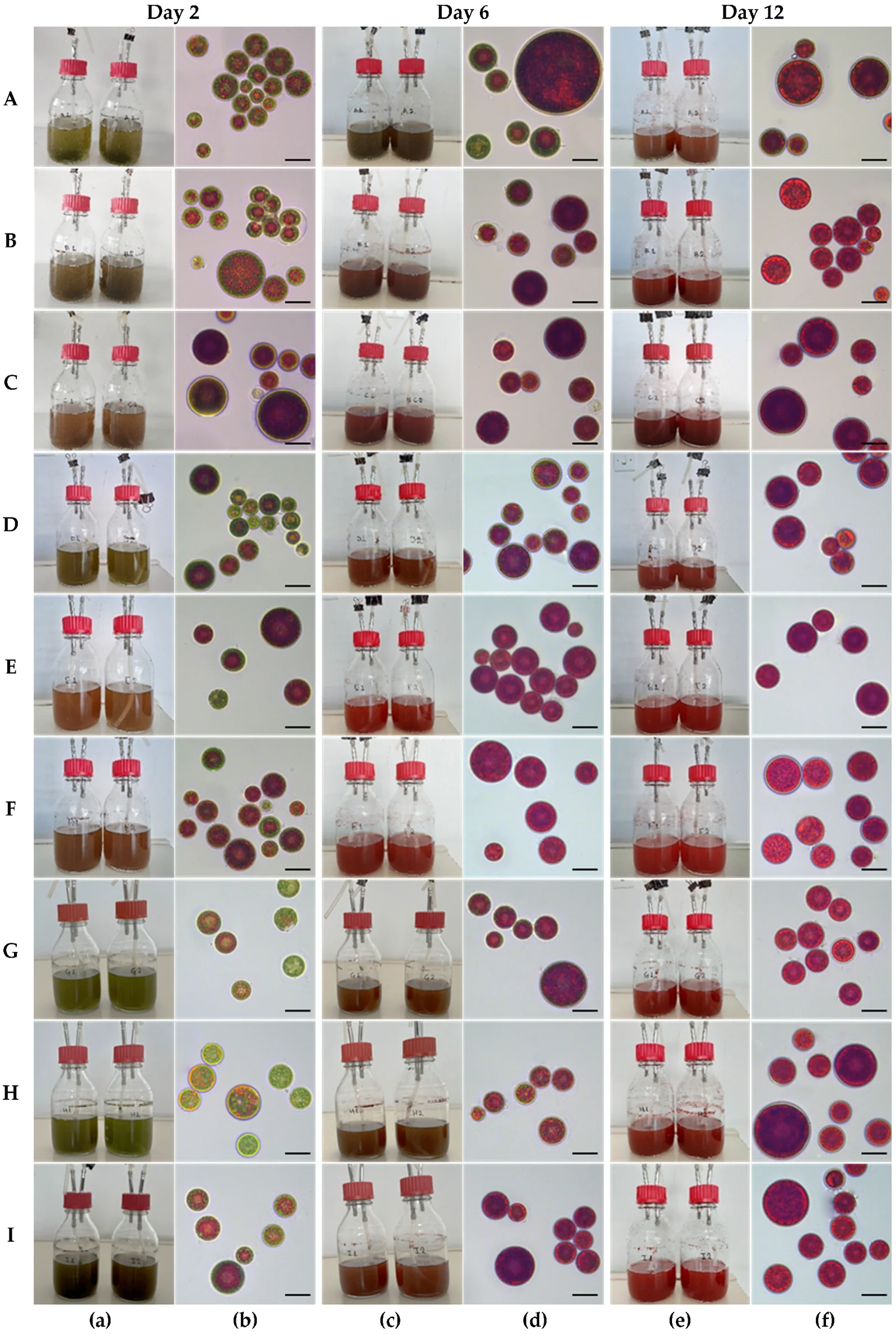
(**a**,**c**,**e**) Macroscopic and (**b**,**d**,**f**) microscopic images of the cultures (A–I) on day 2 (**a**,**b**), day 6 (**c**,**d**) and day 12 (**e**,**f**). Scale bar: 20 μm.

The distinction between cellular and culture-level performance was clearest for cultures C and I (Figure 1d). The comparison illustrates that the tested factor combinations effectively differentiated between two desirable production outcomes: stronger cellular enrichment in Culture C and maximizing volumetric astaxanthin production in Culture I. Since volumetric astaxanthin production is a function of both DCW (g/L) and cellular pigment content (mg/g), applying the highest level of each variable successfully maximizes the total astaxanthin yield (Figure 2c), highlighting a combined effect among temperature, carbon availability and salinity. Although Culture C maximized cellular astaxanthin content, it reached a respective volumetric concentration of 79.7 mg/L. Culture I combined 38.8 mg/g with the highest DCW, yielding the maximum astaxanthin concentration of 96.7 mg/L, outperforming Culture C by 21.4%, whereas Culture A reached only 27.9 mg/L. This low yield of Culture A, exhibiting the minimum astaxanthin concentration when the lowest levels of the three studied factors were applied (Figure 1d), demonstrates that the baseline stressors of nutrient starvation and intense light are not always adequate stressors for increasing astaxanthin production, but the appropriate temperature–CO_2_–salinity combination can lead to optimal integration of cellular enrichment and biomass formation into high volumetric astaxanthin accumulation. Macroscopic and microscopic observations qualitatively supported the astaxanthin time course (Figure A1, Appendix A). By day 2, all cultures showed hematocyst formation and intracellular orange–red pigmentation, which was fully developed by day 12, although to different extents (compared to the status of the cultures on day 0 depicted in Figure 1c,d).

Comparison of the present results with the relevant literature-reported data indicates that the 12-day endpoint values achieved in this study are highly competitive and stand out among published photoautotrophic induction studies, although they do not represent the highest values reported to date. In particular, following 10 days of sudden nitrogen starvation, Samhat et al. [41] reported a cellular astaxanthin content of 32.1 mg/g, 1.59 g/L biomass, and a volumetric astaxanthin concentration of approximately 50 mg/L, while the cell-transformation strategy combined with initial-biomass loading decisions reported by Li et al. [42] produced 33.35 mg/g cellular astaxanthin at 2.62 g/L biomass and 87.35 mg/L volumetric astaxanthin. In the present study, Culture I numerically approached or exceeded all three performance indicators, reaching 38.8 mg/g, 2.49 g/L, and 96.7 mg/L, respectively. As these studies differed considerably in cultivation configuration, light regime, initial biomass concentration, and the stress induction strategy, these differences cannot be entirely attributed to the effects of temperature, CO_2_ supplementation and salinity. Higher production levels (56.87 mg/g cellular astaxanthin, 3.77 g/L biomass, and 216.56 mg/L volumetric astaxanthin) have nevertheless been achieved under a pH-controlled cultivation system with regulated carbon and nitrogen supply, operated under higher irradiance (480 μmol/m^2^/s) [36], while induction strategies based on resveratrol/catechol and prolonged phosphate limitation generated markedly higher cellular astaxanthin contents of 7.09 and 7.12% wt., respectively, but considerably lower volumetric concentrations of 42.99 and 27.0 mg/L because biomass formation remained limited [17,43].

Collectively, these comparisons indicate that the cultivation strategies proposed in the present study consistently provide a favorable balance between cellular astaxanthin accumulation and sustained biomass production, while offering considerable potential for further improvement under enhanced illumination. Since photoautotrophic red-stage cultivation requires an effective compromise between pigment enrichment and biomass productivity, the orthogonal structure of L9 design proved effective in decoupling this trade-off, separating the operational factors that primarily drive cellular astaxanthin content (mg/g) from those governing volumetric concentration (mg/L). In this regard, the performance achieved by Culture I under complete nitrogen and phosphorus deprivation represents a quantitatively robust benchmark for future cultivation strategies aiming to simultaneously enhance biomass formation and astaxanthin accumulation.

### 2.2. Inductive-Stage Shifts in Biochemical Composition

**Figure 3:**
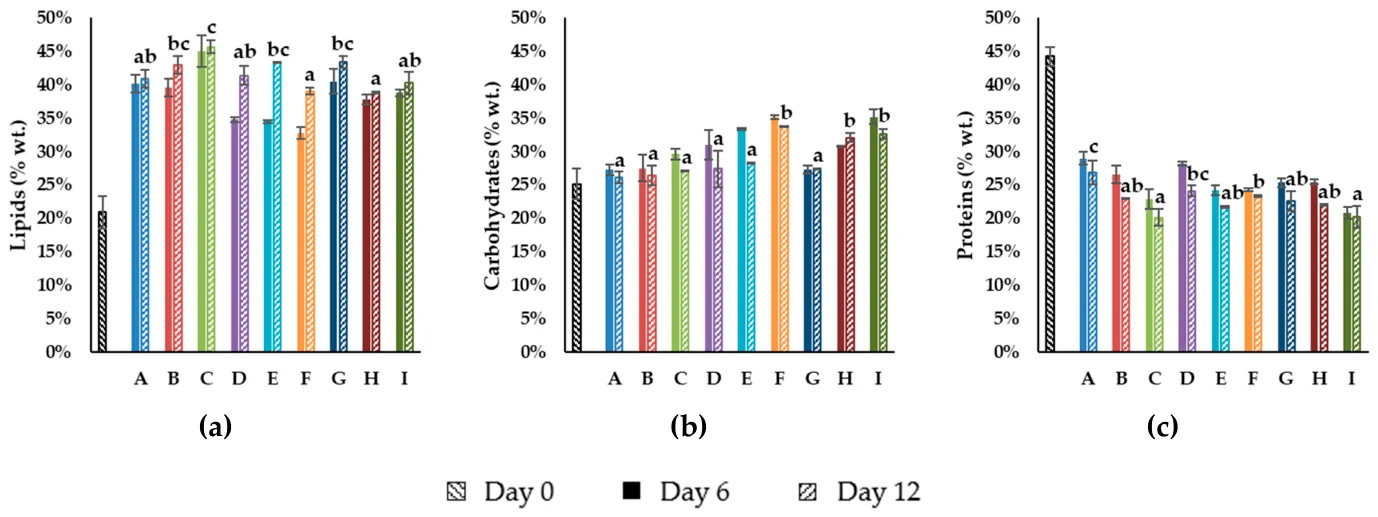
Accumulation of (**a**) lipids; (**b**) carbohydrates and (**c**) proteins. Error bars represent the standard deviation of the two biological replicates. Responses not sharing a letter are statistically different as detected by Tukey’s pairwise comparisons at *p* < 0.05 (performed only for biomolecular contents of day 12).

The inoculum contained 20.9% wt. lipids, 25.1% wt. carbohydrates, and 44.2% wt. proteins. By day 6, protein content decreased to 20.8–29.0% wt., whereas carbohydrates and lipids increased to 27.2–35.2% wt. and 32.8–45.0% wt., respectively (Figure 3). By day 12 proteins declined further to 20.1–26.9% wt. and lipids reached 38.8–45.7% wt.; carbohydrates decreased from day 6 in every culture (26.1–33.9% wt.) but remained above the inoculum level. Most protein degradation and approximately 83% of the mean net lipid increase occurred by day 6, identifying the first half of the induction strategy as the main period of compositional change; however, final composition remained condition-dependent.

**Figure 4:**
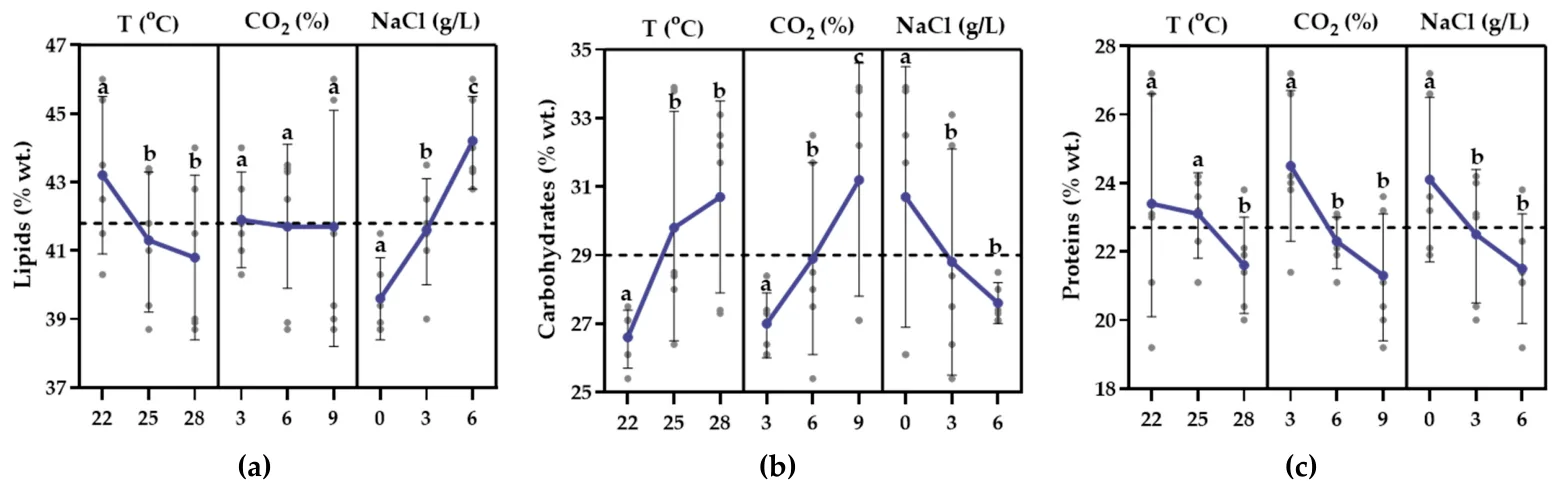
Main effects plots for (**a**) lipid accumulation; (**b**) carbohydrate accumulation; and (**c**) protein accumulation. Gray dots represent the real values of the independent biological cultures, while purple dots represent the mean value of the 6 cultures sharing the same level. Error bars represent the 95% Confidence Interval (CI). The dashed line represents the total mean of the 18 independent biological cultures. Levels of each factor that do not share a letter are statistically different as detected by Tukey’s pairwise comparisons at *p* < 0.05.

Culture C, together with Cultures G and E, achieved the highest observed lipid contents (45.7, 43.4 and 43.3% wt., respectively). Those cultures were exposed to the highest level of salinity (6 g/L NaCl), whereas Cultures H and F, exposed to zero salinity, produced the lowest observed lipid contents of 38.8 and 39.0% wt. (Figure 3a). The highest carbohydrate contents (Figure 3b) were observed in F, I, and H (33.9, 32.6, and 32.1% wt.), all classified among the most productive cultures (Figure 1b). Consequently, it is observed that carbohydrates are tightly corelated with biomass production, with Cultures F, H, and I exhibiting maximum carbohydrate contents alongside peak biomass, whereas lipids are linked with astaxanthin synthesis, with Cultures C, E, and G accumulating maximum lipid and astaxanthin contents. Although simultaneous increases in astaxanthin and lipids are frequently reported during the inductive stage of *H. lacustris* [8,13], the present results suggest that this parallel accumulation is strictly mediated by salinity (Figure 2b and Figure 4a), which appears to stimulate lipid accumulation, as a protective response against stress [8]. When comparing cultures grown at 22 °C (A–C) against those grown at 25 and 28 °C (D–F and G–I) it is observed that lower temperatures favored lipid synthesis over carbohydrates (Figure 3a,b). In contrast, cultures supplied with 9% CO_2_ (C, F, I) accumulated more carbohydrates than cultures with 3 and 6% CO_2_ (A, D, G, and B, E, H, respectively), a trend that was not observed in lipid accumulation, indicating that inorganic carbon predominantly influenced carbohydrate accumulation rather than lipid formation. These trends are clearly reflected in the corresponding main effects plots, where temperature and CO_2_ contributed equally through a positive, dose-dependent relationship in carbohydrate content (Figure 4b) but did not promote lipid accumulation (Figure 4a). Therefore, in the absence of salinity stress, high carbon availability and elevated temperatures preferentially upregulate carbohydrate biosynthesis. Finally, the highest protein content (Figure 3c) was observed in Culture A (26.9% wt.), while the lowest were observed in Cultures C and I (20.1 and 20.2% wt.), exhibiting a negative dose-dependent relationship with studied factors (Figure 4c). However, it exhibited the smallest variation, likely because nitrogen deprivation during the transition to the inductive stage represents the primary determinant of protein degradation and cellular reorganization. Thus, while nitrogen starvation establishes the overall biochemical state of aplanospores, temperature, CO_2_ availability, and salinity serve as secondary modulators that fine-tune carbon allocation among astaxanthin, lipids and carbohydrates.

The decline in protein content is consistent with degradation and reorganization of nitrogen-rich cellular components and is conventionally accompanied by enhanced carbohydrate and lipid biosynthesis, typically under nitrogen starvation [16,44]. Carbohydrate content increased through day 6 and then declined, while lipid content continued to rise. A similar temporal pattern was reported for nitrogen-starved *H. lacustris*, in which rapid carbohydrate accumulation occurred as an immediate response to stress, but later declined as cellular carbon was redirected into fatty acid formation [21,45]. Lipid accumulation is particularly relevant to astaxanthin deposition because, during the inductive stage of *H. lacustris*, astaxanthin is predominantly esterified with fatty acids and sequestered in triacylglycerol (TAG)-rich cytosolic lipid droplets [8,46,47]. Nevertheless, TAG accumulation can also occur in the absence of substantial astaxanthin biosynthesis, indicating that the two processes are coordinated but not obligatorily coupled [48]. The present results exhibited a similar overall, although not identical, relationship. Culture C ranked first for both lipid and cellular astaxanthin contents; however, Culture G ranked second in lipid content but only fourth in cellular astaxanthin content, whereas Culture I ranked third in astaxanthin and seventh in total lipid content. This discrepancy is consistent with the fact that total lipids include both storage and membrane fractions, whereas astaxanthin esters occur mainly in the TAG-rich storage lipid compartment [49]. Total lipid content should therefore not be used as a direct indicator of cellular astaxanthin accumulation.

### 2.3. Model Evaluation and Validation

The main indicators of Analysis of Variance (ANOVA) such as *p*-values, estimated factor contributions and model-fit statistics for all studied responses are presented in Table 1. The analysis of the DCW susceptibility to changes in the explored cultivation parameters revealed that CO_2_ availability was the primary driver of biomass accumulation with 58.4% contribution, followed by temperature, while salinity exerted the least statistically significant effect. Regarding astaxanthin content (mg/g), salinity emerged as the most significant factor accounting for 69.3% of the total contribution, followed by CO_2_ concentration, with temperature exhibiting the least significant effect. Conversely, the ranking between salinity and CO_2_ reversed on the final volumetric astaxanthin concentration (mg/L) with CO_2_ availability having the dominant contribution of 48.1%, and with temperature again having the least significant effect. The analysis of biochemical biomass composition demonstrated that all evaluated factors significantly influenced cellular carbon allocation. Carbohydrate content was significantly influenced by all parameters, with temperature and CO_2_ availability contributing equally. Protein accumulation under the common N-depleted environment was likewise significantly affected by all factors, in which CO_2_ concentration was the principal driver (45.5% contribution). In contrast, lipid accumulation was governed predominantly by salinity (70.8% contribution) and temperature, whereas carbon availability (among the studied levels) was statistically insignificant.

**Figure 5:**
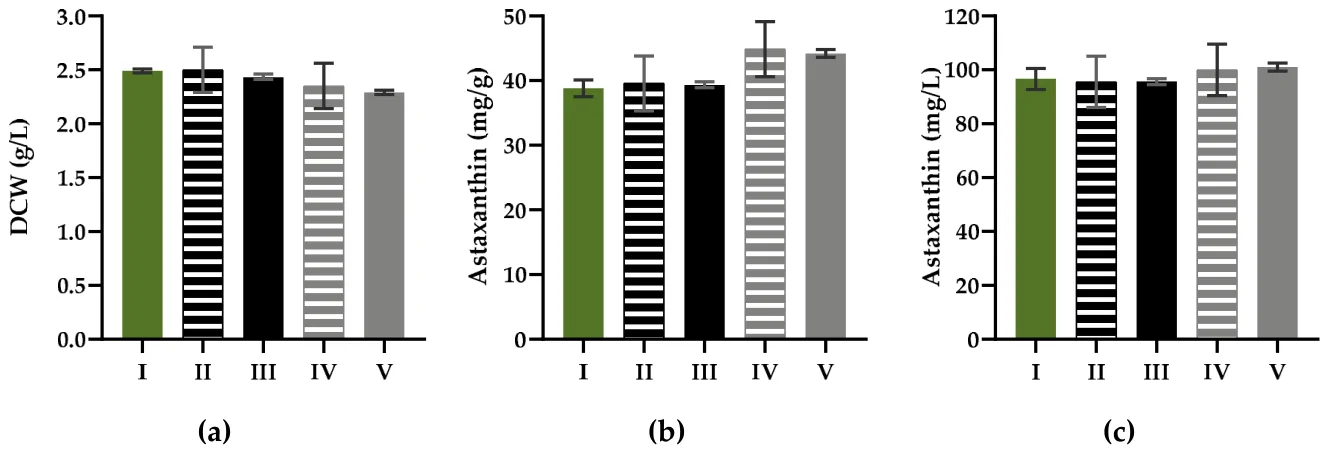
Comparative results of (**a**) biomass production; (**b**) astaxanthin content and (**c**) astaxanthin volumetric production. I: Culture I—main experiment; II: Culture I—predicted response; III: Culture I—repeated experiment; IV: optimal culture—predicted response; and V: optimal culture—experimental response. Error bars represent upper and lower limits of experimental values and 95% prediction interval for the predicted values.

ANOVA confirmed the statistical robustness and high accuracy of the developed models, with coefficients of determination (R^2^) exceeding 90% across all evaluated responses except protein content. Furthermore, predictive R^2^ values surpassed 80% for these same responses, demonstrating strong predictive validity. Model predictions for unperformed parameter combinations are presented in Appendix B. Model fidelity was verified across all 18 biological observations, through high coefficients of determination (R^2^ = 0.9984 − 0.9998) in observed-vs-predicted scatter plots (Figure S1). Anderson–Darling tests did not identify statistically significant deviation from residual normality for any fitted response (*p* = 0.055 − 0.718; Table S1), with normal probability plots of standardized residuals adhering closely to theoretical distributions (Figure S2). Furthermore, residual-versus-fitted assessments showed no pronounced systematic patterns or changes in dispersion (Table S1). Based on the model predictions, the optimal parameter combination for maximizing volumetric astaxanthin production was identified as 28 °C, 9% CO_2_, and 6 g/L NaCl, yielding a predicted maximum concentration of 100.0 mg/L (Appendix B, Table A1). This optimal combination was experimentally validated, together with the best performing Culture I, which was again repeated to evaluate the reproducibility. A comparative analysis of culture performance on the final day between those two conditions is presented in Figure 5 in terms of biomass production, astaxanthin content and volumetric production, while the detailed performance of the cultures throughout the cultivation period is presented in the Supplementary Material (Figure S3).

Notably, the model-predicted values were in strong agreement with the experimental determinations for DCW, cellular astaxanthin content and volumetric production across both evaluated conditions (Figure 5). Specifically, for DCW (Figure 5a), the experimental validation value obtained for the optimal culture (bar V, 2.29 g/L) closely matched the model-predicted response (bar IV, 2.35 g/L), confirming the predictive accuracy of the Taguchi analysis. A similar robust alignment was observed for both cellular astaxanthin content (Figure 5b) and volumetric astaxanthin production (Figure 5c), where the experimental outcomes of the optimal culture (bar V, 44.2 mg/g and 101.0 mg/L) closely approached the predicted values (bar IV, 44.9 mg/g and 100.0 mg/L). The outcomes of bar III, which corresponds to the replication of bar I, demonstrated high reproducibility relative to Culture I. Across all three responses, the experimental validation runs (bars III and V) fell within the 95% prediction interval for the predicted values (bars II and IV).

The above findings suggest that further improvements in astaxanthin production may be achievable under operational conditions beyond the upper limits evaluated in this study, since maximum astaxanthin was produced under the highest level of each factor. However, the absence of a statistically significant difference between medium and high salinity levels (Figure 2c) may indicate that salinity effects approach a saturation threshold, beyond which further increases may compromise overall productivity. Alternatively, the limited response at elevated salinity may reflect insufficient light availability, suggesting that higher irradiance could be required to fully exploit the potential benefits of combined salinity and carbon enrichment.

## 3. Materials and Methods

### 3.1. Microalgal Strain and Culture Medium

The microalgal strain *Haematococcus pluvialis* SAG 34-1b (a synonym for *H. lacustris*) was purchased from the Experimental Phycology and Culture Collection of Algae at the University of Göttingen (EPSAG), Germany. The Optimal Haematococcus Medium (OHM) [50] was used for precultures, while for the main experiment it was modified by totally deducting the nitrogen and phosphorus sources. Precultures were carried out in 1 L Erlenmeyer flasks with 0.5 L working volume in a shaking incubator under the optimal vegetative stage identified previously [37] at 22 °C and 140 rpm, under 100 μmol/m^2^/s illumination intensity with 16/8 h light/dark photoperiod and 0.1 L/min air supply. For the maintenance of the cells in the vegetative phase, precultures were centrifuged and resuspended in fresh OHM every 7 days. This regular recultivation ensured a uniform physiological state and provided consistent inoculums for all experimental runs.

### 3.2. Cultivation System and Culture Conditions

**Figure 6:**
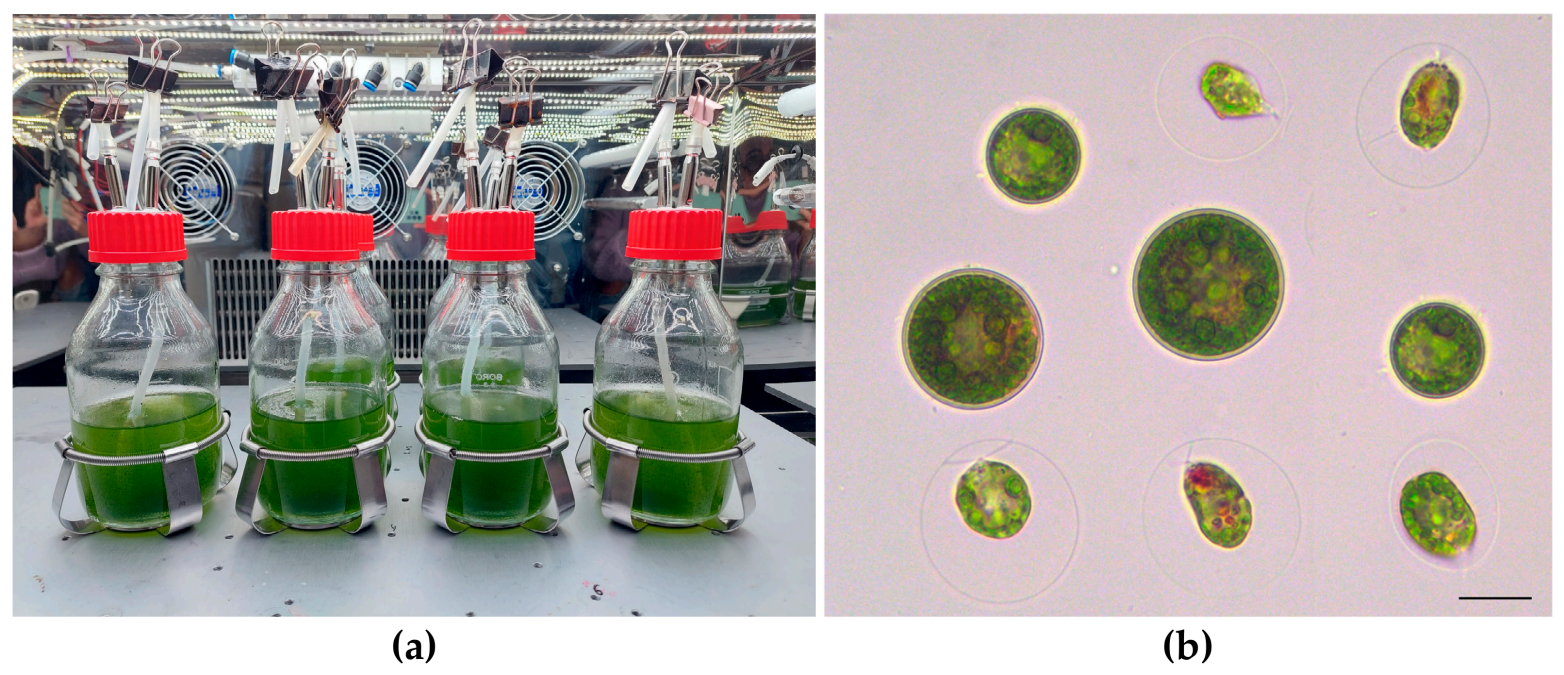
(**a**) Macroscopic and (**b**) microscopic images of the cultures on Day 0. Scale bar: 20 μm.

In this study, the effect of cultivation temperature, CO_2_ composition in the headspace of the closed Duran glass bottles, and salinity of the culture medium were simultaneously investigated with respect to biomass production, intracellular composition and astaxanthin accumulation during the inductive stage of *H. lacustris* cultivation over a 12-day period. The studied levels for the aforementioned factors were 22, 25 and 28 °C for temperature; 3, 6 and 9% *v*/*v* for CO_2_ composition in the headspace; and 0, 3 and 6 g/L NaCl for salinity. All the experiments were conducted in a shaking incubator (BJPX-2012R, BIOBASE, China), equipped with cool-white LED strips, using 500 mL Duran bottles with 300 mL working volume (Figure 6a).

The initial biomass was set at 0.4 g/L to prevent photo-bleaching under high light intensity while avoiding self-shading during early induction. The illumination was continuous with an intensity of 200 μmol/m^2^/s, and the shaking rate was 150 rpm. Duran bottles were aerated once a day with the corresponding mixture of CO_2_-enriched air until saturation and then immediately sealed with cups, ensuring no air exchange with the atmosphere. Saturation was ascertained by measuring the headspace exhaust stream with an exhaust gas analyzer (bBreath4, Bionet, Spain). The composition of each culture’s headspace was monitored daily before air renewal with the exhaust gas analyzer. The contained O_2_ for all cultures exceeded the upper measurable threshold of the analyzer (50% *v*/*v*), while the CO_2_ was totally assimilated (measurements of 0.0% *v*/*v* were recorded). Finally, the pH was checked daily prior to headspace regeneration and adjusted to 7 ± 0.1 (observed fluctuations ranged between 6.0 and 8.0) using 1 M HCl or NaOH solutions.

The implementation of a CO_2_-enriched headspace in a completely sealed experimental setup (i.e., Duran glass bottles) was adopted as a mitigation strategy to minimize carbon losses during the inductive stage. Unlike continuous gas sparging at high CO_2_ levels (up to 10% *v*/*v*), which often leads to low carbon utilization due to short gas–liquid retention time, the closed system retained the supplied carbon. At the same time, this sealed configuration allowed the culture to generate its own oxidative environment. As photosynthesis proceeded, cells consumed CO_2_ and released oxygen, increasing dissolved and headspace oxygen levels. The progressively increasing levels of oxygen were implicitly expected to trigger astaxanthin synthesis as an antioxidant defense. However, to prevent excessive hyperoxia and replenish inorganic carbon, the headspace was renewed once daily with the designated CO_2_-enriched air mixture. This daily headspace refreshment vented the accumulated oxygen and concurrently provided fresh inorganic carbon to sustain the photosynthetic performance.

### 3.3. Taguchi L9 Orthogonal-Array Design

To reduce the total number of experiments needed (helping to sustain consistent conditions and experimental handling across all treatments), a fractional factorial L9 Taguchi design was applied, resulting in the conduction of 9 trials instead of 27 required by the full factorial design. According to the applied Taguchi design the combination of the above factors resulted in the nine experiments presented in the table in Figure 1. The three independent variables were treated as fixed categorical factors across three operational levels. All experimental runs were performed in duplicate (*n* = 2), biologically independent, yielding a total of 18 biological observations. Analytical measurements were conducted in triplicate (r = 3) and averaged within each biological replicate prior to statistical analysis. In this framework, each three-level factor contributes two degrees of freedom (DFs), resulting in six model DFs. With 18 biological observations, the replicated L9 structure provides 11 residual DFs, partitioned into nine pure-error DFs and two lack-of-fit DFs. Two-factor interaction terms were excluded from the model, as complete pairwise interactions are structurally aliased and cannot be independently estimated within an unaugmented L9 orthogonal array.

### 3.4. Analytical Methods

Dry cell weight (DCW) was determined by filtering 2 mL of culture through dry pre-weighed glass fiber filters (Whatman 934-AH, pore size 1.2 μm) that were subsequently washed twice with distilled water, dried at 45 °C for at least 24 h and weighed on a 5-decimal scale (PIONEER PX85, OHAUS, Greifensee, Switzerland). Astaxanthin was determined by adding 2 mL of dimethyl sulfoxide (DMSO) in 1 mg of freeze-dried samples for pigment extraction. The samples were heated to 70 °C for 10 min in a shaking heater (Thermomixer compact 5350, Eppendorf, Hamburg, Germany) and after centrifugation at 5000 rpm for 5 min (150R, MPW MED. INSTRUMENTS, Kraków, Poland) the absorbance of the supernatant was measured at 530 nm on a Vis-spectrophotometer (DR3900, HACH, Berlin, Germany) [51]. Astaxanthin standard (purity 97.9%) was purchased from LGC Standards for the calibration curve at 530 nm. Carbohydrates were measured using the phenol-sulfuric method [52], proteins were determined by the bicinchoninic acid method [53] and lipids were measured with the sulfo-phospho-vanillin method [54]. DCW and astaxanthin content were determined every 2 days, while carbohydrate, protein and lipid contents were measured on days 0, 6 and 12.

### 3.5. Statistical Analysis

Experimental data were analyzed via Analysis of Variance (ANOVA), using a General Linear Model. For each culture response, an additive main-effects formulation was fitted according to the following equation:

Y = μ + α_i_T_i_ + β_j_C_j_ + γ_k_S_k_ + ε (i,j,k = 1, 2 or 3) (1)

where μ is the overall mean; α_i_, β_j_, and γ_k_ are fixed coefficients weighting the effects of the individual factors T_i_, C_j_ and S_k_ (temperature, CO_2_, and salinity) across each of the three levels (i, j, k); and ε is the random error term. Model fidelity and linearity were inspected using observed-vs-predicted response plots. Normality of the residuals was verified visually through normal probability plots and confirmed quantitatively via the Anderson–Darling test (*p* > 0.05). Standardized residuals were inspected to identify observations showing comparatively large deviations from the fitted models. When a factor’s effect was statistically significant, differences among operational factor levels were evaluated using Tukey’s pairwise comparisons at the 95% confidence level (α = 0.05). Model goodness-of-fit and predictive capability were quantified using the coefficient of determination (R^2^), adjusted R^2^ (R^2^_adj_), and predicted R^2^ (R^2^_pred_). R^2^_pred_ was calculated from the prediction error sum of squares (PRESS), corresponding to leave-one-observation-out internal cross-validation.

The Taguchi design, the data analysis (ANOVA), the main effect plots, as well as the statistical model development and derived predictions of process responses were all performed using the Minitab Statistical Software (version 22, Minitab LLC, State College, PA, USA). Based on the statistical analysis of the initial nine biological observations, an optimal combination of culture conditions was predicted to maximize volumetric astaxanthin production. Because this specific combination was not part of the initial experimental matrix, it was experimentally validated and compared against a replicated run of the best-performing initial trial. Both conditions were evaluated in duplicate (*n* = 2) to assess reproducibility, compare relative performance and validate the reliability of the Taguchi model predictions.

## 4. Conclusions

This study successfully demonstrated the feasibility of utilizing a non-aerated, sealed cultivation system with a CO_2_-enriched headspace for cultivating *Haematococcus lacustris* during the inductive stage, and identified the relative influence of temperature, CO_2_ concentration and salinity on efficient astaxanthin production. Taguchi analysis revealed that salinity is the strongest driver for this metabolic shift, temperature regulates the rate of this shifting under stress, and inorganic carbon availability determines whether cells can maintain enough metabolic activity to sustain both viability and pigment accumulation. Statistical analysis confirmed that all applied stress factors significantly affected volumetric astaxanthin production, demonstrating that their application as complementary stressors alongside nutrient limitation (i.e., nitrogen and phosphorus) and high irradiance can regulate and further improve photoautotrophic inductive-stage performance. The combination of increased salinity with sufficient carbon supply forced the microalga to redirect its metabolism towards lipid–astaxanthin biosynthesis as part of the cellular antioxidant response. The experimental combination of high temperature and CO_2_ with medium salinity resulted in a peak volumetric concentration of 96.7 mg/L in Experiment I, while statistical model prediction suggested the implementation of the highest value of all factors to achieve a maximum astaxanthin production of 100.0 mg/L. This predicted maximum was experimentally validated (101.0 mg/L), confirming the robustness and predictive accuracy of the Taguchi analysis. Ultimately, this non-aerated system presents a highly promising, low-energy configuration for cost-effective astaxanthin production with reduced/zero carbon losses. The results also provide a practical basis for optimizing environmental stress combinations during the inductive stage and for further improving process performance through enhanced illumination and refinement of operational conditions.

## Figures and Tables

**Table 1:** ANOVA *p*-values, estimated factor contributions, and model-fit statistics for biomass concentration, astaxanthin content and volumetric concentration, and biochemical composition of *H. lacustris* on day 12. For each response, the factor with the highest estimated contribution is shown in bold.

Response	Temperature (°C)		CO_2_ (% *v*/*v*)		Salinity (g/L NaCl)		Model Fit		
	*p*-Value	Contrib. (%)	*p*-Value	Contrib. (%)	*p*-Value	Contrib. (%)	*R*^2^ (%)	*R*^2^ _(adj)_ (%)	*R*^2^ _(pred)_ (%)
Dry cell weight (g/L)	0.000	33.2	0.000	**58.4**	0.023	4.2	95.71	93.37	88.52
Astaxanthin (mg/g)	0.007	5.1	0.000	22.1	0.000	**69.3**	96.54	94.65	90.74
Astaxanthin (mg/L)	0.000	16.4	0.000	**48.1**	0.000	33.1	97.57	96.24	93.49
Carbohydrates (% wt.)	0.000	**39.2**	0.000	37.0	0.001	20.1	96.29	94.27	90.07
Proteins (% wt.)	0.013	14.3	0.000	**45.5**	0.001	28.1	87.97	81.40	67.78
Lipids (% wt.)	0.001	21.6	0.860	0.2	0.000	**70.8**	92.67	88.67	80.37

Percentage contribution was calculated as (factor’s adjusted sum of squares/total sum of squares) × 100. An investigated factor was considered to have a statistically significant effect on a response at *p* < 0.05.

## Data Availability

The original contributions presented in this study are included in the article/Supplementary Material. Further inquiries can be directed to the corresponding author.

## Supplementary Materials

The following supporting information can be downloaded at: https://www.mdpi.com/article/10.3390/md24100350/s1, Figure S1. Ob-served-vs-Predicted plot for (a) Biomass; (b) Astaxanthin content; (c) Astaxanthin concentration; (d) Carbohydrate accumulation; (e) Protein accumulation and (f) Lipid accumulation. Figure S2. Normal probability plots of residuals for (a) Biomass; (b) Astaxanthin content; (c) Astaxanthin concentration; (d) Carbohydrate accumulation; (e) Protein accumulation and (f) Lipid accumulation. Models were fitted using 18 independent biological culture observations. Figure S3. Production of (a) Total Biomass; (b) Astaxanthin content and (c) Astaxanthin concentration. I: Culture I—main experiment; III: Culture I—repeated experiment and V: optimal culture—experimental response. Error bars represent the standard deviation of the two biological replicates. Table S1. Summary of residual diagnostics for the additive main-effects models fitted to the day-12 responses.

## Appendix B

The data produced from model predictions are presented in Table A1. The data include results for both non-conducted and conducted experimental trials.

**Table A1:** Model predictions for DCW, astaxanthin content and astaxanthin concentration. Values in brackets correspond to experimentally measured data.

Temperature (°C)	CO_2_ (% *v*/*v*)	NaCl (g/L)	DCW (g/L)	Astaxanthin (mg/g)	Astaxanthin (mg/L)
22	3	0	1.46 (1.45)	20.0 (19.2)	26.8 (27.9)
22	3	3	1.56	28.7	46.8
22	3	6	1.42	34.0	51.2
22	6	0	1.94	23.9	47.7
22	6	3	2.04 (2.09)	32.6 (33.0)	67.7 (68.8)
22	6	6	1.90	37.9	72.1
22	9	0	1.96	28.0	57.5
22	9	3	2.07	36.6	77.6
22	9	6	1.92 (1.88)	41.9 (42.3)	81.9 (79.7)
25	3	0	1.66	23.6	38.6
25	3	3	1.76 (1.73)	32.3 (32.6)	58.6 (56.3)
25	3	6	1.61	37.6	63.0
25	6	0	2.14	27.5	59.5
25	6	3	2.24	36.2	79.5
25	6	6	2.09 (2.09)	41.5 (40.7)	83.9 (85.0)
25	9	0	2.16 (2.21)	31.6 (32.0)	69.3 (70.5)
25	9	3	2.27	40.2	89.4
25	9	6	2.12	45.5	93.8
28	3	0	1.89	22.9	44.8
28	3	3	1.99	31.6	64.8
28	3	6	1.84 (1.89)	36.9 (37.3)	69.2 (70.4)
28	6	0	2.37 (2.33)	26.9 (27.2)	65.7 (63.4)
28	6	3	2.47	35.5	85.7
28	6	6	2.33	40.8	90.1
28	9	0	2.39	30.9	75.6
28	9	3	2.50 (2.49)	39.6 (38.8)	95.6 (96.7)
**28**	**9**	**6**	**2.35**	**44.9**	**100.0**

## References

[1] Dolganyuk V., Belova D., Babich O., Prosekov A., Ivanova S., Katserov D., Patyukov N., Sukhikh S. (2020). Microalgae: A Promising Source of Valuable Bioproducts. Biomolecules.

[2] Mularczyk M., Michalak I., Marycz K. (2020). Astaxanthin and Other Nutrients from Haematococcus Pluvialis—Multifunctional Applications. Mar. Drugs.

[3] Dutta S., Kumar S.P.J., Banerjee R. (2023). A Comprehensive Review on Astaxanthin Sources, Structure, Biochemistry and Applications in the Cosmetic Industry. Algal Res..

[4] Tominaga K., Hongo N., Karato M., Yamashita E. (2012). Cosmetic Benefits of Astaxanthin on Humans Subjects. Acta Biochim. Pol..

[5] Nakada T., Ota S. (2016). What Is the Correct Name for the Type of Haematococcus Flot. (Volvocales, Chlorophyceae)?. Taxon.

[6] Li X., Wang X., Duan C., Yi S., Gao Z., Xiao C., Agathos S.N., Wang G., Li J. (2020). Biotechnological Production of Astaxanthin from the Microalga Haematococcus Pluvialis. Biotechnol. Adv..

[7] Oslan S.N.H., Shoparwe N.F., Yusoff A.H., Rahim A.A., Chang C.S., Tan J.S., Oslan S.N., Arumugam K., Ariff A.B., Sulaiman A.Z. (2021). A Review on Haematococcus Pluvialis Bioprocess Optimization of Green and Red Stage Culture Conditions for the Production of Natural Astaxanthin. Biomolecules.

[8] Chen G., Wang B., Han D., Sommerfeld M., Lu Y., Chen F., Hu Q. (2015). Molecular Mechanisms of the Coordination between Astaxanthin and Fatty Acid Biosynthesis in Haematococcus Pluvialis (Chlorophyceae). Plant J..

[9] Aflalo C., Meshulam Y., Zarka A., Boussiba S. (2007). On the Relative Efficiency of Two- vs. One-stage Production of Astaxanthin by the Green Alga Haematococcus Pluvialis. Biotechnol. Bioeng..

[10] Del Río E., Acién F.G., García-Malea M.C., Rivas J., Molina-Grima E., Guerrero M.G. (2008). Efficiency Assessment of the One-step Production of Astaxanthin by the Microalga Haematococcus Pluvialis. Biotechnol. Bioeng..

[11] Boussiba S. (2000). Carotenogenesis in the Green Alga Haematococcus Pluvialis: Cellular Physiology and Stress Response. Physiol. Plant..

[12] Li Y., Sommerfeld M., Chen F., Hu Q. (2008). Consumption of Oxygen by Astaxanthin Biosynthesis: A Protective Mechanism against Oxidative Stress in Haematococcus Pluvialis (Chlorophyceae). J. Plant Physiol..

[13] Wang B., Zarka A., Trebst A., Boussiba S. (2003). Astaxanthin Accumulation in Haematococcus Pluvialis (Chlorophyceae) as an Active Photoprotective Process Under High Irradiance. J. Phycol..

[14] Qiu B., Li Y. (2006). Photosynthetic Acclimation and Photoprotective Mechanism of Haematococcus Pluvialis (Chlorophyceae) during the Accumulation of Secondary Carotenoids at Elevated Irradiation. Phycologia.

[15] Sathiyavahisan L.P., Lakshmi Narasimhan A., Mahadi R., Kim S., Christabel C., Yu H., Kim Y.-E., Oh Y.-K. (2023). Rapid Induction of Astaxanthin in Haematococcus Lacustris by Mild Electric Stimulation. Appl. Sci..

[16] Recht L., Töpfer N., Batushansky A., Sikron N., Gibon Y., Fait A., Nikoloski Z., Boussiba S., Zarka A. (2014). Metabolite Profiling and Integrative Modeling Reveal Metabolic Constraints for Carbon Partitioning under Nitrogen Starvation in the Green Algae Haematococcus Pluvialis. J. Biol. Chem..

[17] Liyanaarachchi V.C., Nishshanka G.K.S.H., Premaratne R.G.M.M., Ariyadasa T.U., Nimarshana P.H.V., Malik A. (2020). Astaxanthin Accumulation in the Green Microalga Haematococcus Pluvialis: Effect of Initial Phosphate Concentration and Stepwise/Continuous Light Stress. Biotechnol. Rep..

[18] Zhang W., Zhou X., Zhang Y., Cheng P., Ma R., Cheng W., Chu H. (2018). Enhancing Astaxanthin Accumulation in Haematococcus Pluvialis by Coupled Light Intensity and Nitrogen Starvation in Column Photobioreactors. J. Microbiol. Biotechnol..

[19] Giannelli L., Yamada H., Katsuda T., Yamaji H. (2015). Effects of Temperature on the Astaxanthin Productivity and Light Harvesting Characteristics of the Green Alga Haematococcus Pluvialis. J. Biosci. Bioeng..

[20] Jin C., You J., Zhou Z., Liu Q., Zhou X. (2025). Novel Insights into Saline Stress on Photosynthetic Activity and Astaxanthin Production of Haematococcus Pluvialis. J. Oceanol. Limnol..

[21] Li Q., You J., Qiao T., Zhong D., Yu X. (2022). Sodium Chloride Stimulates the Biomass and Astaxanthin Production by Haematococcus Pluvialis via a Two-Stage Cultivation Strategy. Bioresour. Technol..

[22] Chekanov K., Schastnaya E., Solovchenko A., Lobakova E. (2017). Effects of CO_2_ Enrichment on Primary Photochemistry, Growth and Astaxanthin Accumulation in the Chlorophyte Haematococcus Pluvialis. J. Photochem. Photobiol. B.

[23] Christian D., Zhang J., Sawdon A.J., Peng C.-A. (2018). Enhanced Astaxanthin Accumulation in Haematococcus Pluvialis Using High Carbon Dioxide Concentration and Light Illumination. Bioresour. Technol..

[24] Zheng Q., Martin G.J.O., Kentish S.E. (2016). Energy Efficient Transfer of Carbon Dioxide from Flue Gases to Microalgal Systems. Energy Environ. Sci..

[25] Sarada R., Bhattacharya S., Bhattacharya S., Ravishankar G.A. (2002). A Response Surface Approach for the Production of Natural Pigment Astaxanthin from Green Alga, Haematococcus Pluvialis: Effect of Sodium Acetate, Culture Age, and Sodium Chloride. Food Biotechnol..

[26] Niizawa I., Espinaco B.Y., Leonardi J.R., Heinrich J.M., Sihufe G.A. (2018). Enhancement of Astaxanthin Production from Haematococcus Pluvialis under Autotrophic Growth Conditions by a Sequential Stress Strategy. Prep. Biochem. Biotechnol..

[27] Xing H., Zhao Y., Li T., Han B., Zhao P., Yu X. (2022). Enhancing Astaxanthin and Lipid Coproduction in Haematococcus Pluvialis by the Combined Induction of Plant Growth Regulators and Multiple Stresses. Bioresour. Technol..

[28] Harker M., Tsavalos A.J., Young A.J. (1995). Use of Response Surface Methodology to Optimise Carotenogenesis in the Microalga, Haematococcus Pluvialis. J. Appl. Phycol..

[29] Wang L., Dai Y., Huang X. (2024). Optimization of Astaxanthin Accumulation in a New Haematococcus Lacustris (CZJ) Strain from China via Response Surface Methodology. J. Appl. Phycol..

[30] Pereira S., Otero A. (2020). Haematococcus Pluvialis Bioprocess Optimization: Effect of Light Quality, Temperature and Irradiance on Growth, Pigment Content and Photosynthetic Response. Algal Res..

[31] Hong M.-E., Hwang S.K., Chang W.S., Kim B.W., Lee J., Sim S.J. (2015). Enhanced Autotrophic Astaxanthin Production from Haematococcus Pluvialis under High Temperature via Heat Stress-Driven Haber–Weiss Reaction. Appl. Microbiol. Biotechnol..

[32] Wan M., Zhang J., Hou D., Fan J., Li Y., Huang J., Wang J. (2014). The Effect of Temperature on Cell Growth and Astaxanthin Accumulation of Haematococcus Pluvialis during a Light-Dark Cyclic Cultivation. Bioresour. Technol..

[33] Li Q., Zhao Y., Ding W., Han B., Geng S., Ning D., Ma T., Yu X. (2021). Gamma-Aminobutyric Acid Facilitates the Simultaneous Production of Biomass, Astaxanthin and Lipids in Haematococcus Pluvialis under Salinity and High-Light Stress Conditions. Bioresour. Technol..

[34] Yang H.E., Yu B.S., Sim S.J. (2023). Enhanced Astaxanthin Production of Haematococcus Pluvialis Strains Induced Salt and High Light Resistance with Gamma Irradiation. Bioresour. Technol..

[35] Yang S., Zhao W., Mou H., Sun H. (2021). Improving Astaxanthin Production of Haematococcus Pluvialis by an Efficient Fed-Batch Strategy in a Photobioreactor. Algal Res..

[36] Ma R., Tao X., Chua E.T., Ho S.H., Shi X., Liu L., Xie Y., Chen J. (2022). Enhancing Astaxanthin Production in Haematococcus Pluvialis QLD by a PH Steady NaHCO_3_-CO_2_-C/NH_4_Cl-N Culture System. Algal Res..

[37] Samara C., Biziouras A., Papapanagiotou G., Chatzidoukas C. (2025). Extending the Green Stage of Haematococcus Pluvialis as a Crucial Precursor for Efficient Astaxanthin Production: Optimization via Taguchi Design. Biochem. Eng. J..

[38] Li K., Cheng J., Lu H., Yang W., Zhou J., Cen K. (2017). Transcriptome-based analysis on carbon metabolism of *Haematococcus pluvialis* mutant under 15% CO_2_. Bioresour. Technol..

[39] Jiang H., Lin X., Xuan W. (2017). Effects of Salt Stress on Astaxanthin Accumulation, Gene Expression of Astaxanthin Synthesis-Related Enzymes, and Antioxidant Indices in Haematococcus Pluvialis. J. Fish. Sci. China.

[40] Gao Z., Meng C., Chen Y.C., Ahmed F., Mangott A., Schenk P.M., Li Y. (2015). Comparison of Astaxanthin Accumulation and Biosynthesis Gene Expression of Three Haematococcus Pluvialis Strains upon Salinity Stress. J. Appl. Phycol..

[41] Samhat K., Kazbar A., Takache H., Ismail A., Pruvost J. (2023). Influence of Light Absorption Rate on the Astaxanthin Production by the Microalga Haematococcus Pluvialis during Nitrogen Starvation. Bioresour. Bioprocess..

[42] Li F., Cai M., Lin M., Huang X., Wang J., Ke H., Wang C., Zheng X., Chen D., Yang S. (2020). Enhanced Biomass and Astaxanthin Production of Haematococcus Pluvialis by a Cell Transformation Strategy with Optimized Initial Biomass Density. Mar. Drugs.

[43] Qiu J.-F., Yang Y.-C., Li R.-Y., Jiao Y.-H., Mou J.-H., Yang W.-D., Lin C.S.K., Li H.-Y., Wang X. (2024). Synergistic and Stepwise Treatment of Resveratrol and Catechol in Haematococcus Pluvialis for the Overproduction of Biomass and Astaxanthin. Biotechnol. Biofuels Bioprod..

[44] Lu Z., Zheng L., Liu J., Dai J., Song L. (2019). A Novel Fed-Batch Strategy to Boost Cyst Cells Production Based on the Understanding of Intracellular Carbon and Nitrogen Metabolism in Haematococcus Pluvialis. Bioresour. Technol..

[45] Recht L., Zarka A., Boussiba S. (2012). Patterns of Carbohydrate and Fatty Acid Changes under Nitrogen Starvation in the Microalgae Haematococcus Pluvialis and Nannochloropsis Sp.. Appl. Microbiol. Biotechnol..

[46] Zhekisheva M., Boussiba S., Khozin-Goldberg I., Zarka A., Cohen Z. (2002). Accumulation of Oleic Acid in Haematococcus Pluvialis (Chlorophyceae) under Nitrogen Starvation or High Light Is Correlated with That of Astaxanthin Esters. J. Phycol..

[47] Wayama M., Ota S., Matsuura H., Nango N., Hirata A., Kawano S. (2013). Three-Dimensional Ultrastructural Study of Oil and Astaxanthin Accumulation during Encystment in the Green Alga Haematococcus Pluvialis. PLoS ONE.

[48] Zhekisheva M., Zarka A., Khozin-Goldberg I., Cohen Z., Boussiba S. (2005). Inhibition of Astaxanthin Synthesis under High Irradiance Does Not Abolish Triacylglycerol Accumulation in the Green Alga Haematococcus Pluvialis (Chlorophyceae). J. Phycol..

[49] Gwak Y., Hwang Y., Wang B., Kim M., Jeong J., Lee C.-G., Hu Q., Han D., Jin E. (2014). Comparative Analyses of Lipidomes and Transcriptomes Reveal a Concerted Action of Multiple Defensive Systems against Photooxidative Stress in Haematococcus Pluvialis. J. Exp. Bot..

[50] Fábregas J., Domínguez A., Regueiro M., Maseda A., Otero A. (2000). Optimization of Culture Medium for the Continuous Cultivation of the Microalga Haematococcus Pluvialis. Appl. Microbiol. Biotechnol..

[51] Li Y., Miao F., Geng Y., Lu D., Zhang C., Zeng M. (2012). Accurate Quantification of Astaxanthin from Haematococcus Crude Extract Spectrophotometrically. Chin. J. Oceanol. Limnol..

[52] DuBois M., Gilles K.A., Hamilton J.K., Rebers P.A., Smith F. (1956). Colorimetric Method for Determination of Sugars and Related Substances. Anal. Chem..

[53] He F. (2011). BCA (Bicinchoninic Acid) Protein Assay. Bio-Protoco.

[54] Mishra S.K., Suh W.I., Farooq W., Moon M., Shrivastav A., Park M.S., Yang J.-W. (2014). Rapid Quantification of Microalgal Lipids in Aqueous Medium by a Simple Colorimetric Method. Bioresour. Technol..

