# Supplementary material for: Multi-Stress Induction of Astaxanthin Accumulation in Haematococcus lacustris: A Taguchi-Designed Experimental Study
Source: Mar Drugs. 2026 Oct 4;24(10):350. doi: 10.3390/md24100350 (PMC13706114; doi:10.3390/md24100350)
Supplement: Supplementary file 1 [file marinedrugs-24-00350-s001.zip › marinedrugs-4504508-supplementary.pdf]

marine drugs

Article

Multi-stress induction of astaxanthin accumulation in *Haematococcus lacustris:* a Taguchi-designed experimental study

Christina Samara, Despoina Samolada, Evangelia Matzarli, Georgia Papapanagiotou and Christos Chatzidoukas *

Laboratory of Biochemical and Biotechnological Processes (LB²P), Department of Chemical Engineering, Aristotle University of Thessaloniki (AUTH); 54124, Thessaloniki, Greece

* Correspondence: chatzido@auth.gr

Supplementary Material

1. Model fidelity

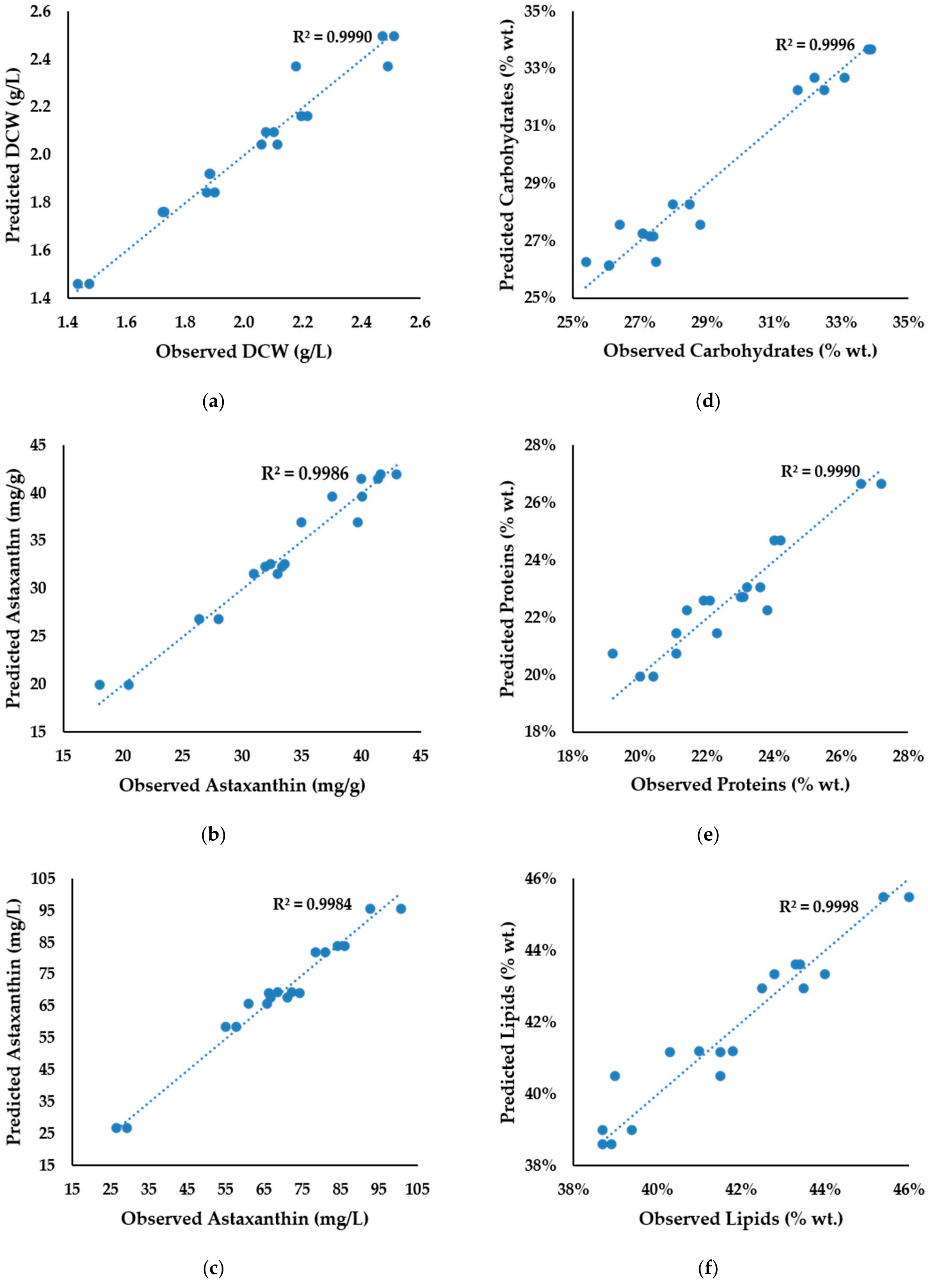

Figure S1. Observed-vs-Predicted plot for (a) Biomass; (b) Astaxanthin content; (c) Astaxanthin concentration; (d) Carbohydrate accumulation; (e) Protein accumulation and (f) Lipid accumulation.

2. Residuals diagnostics

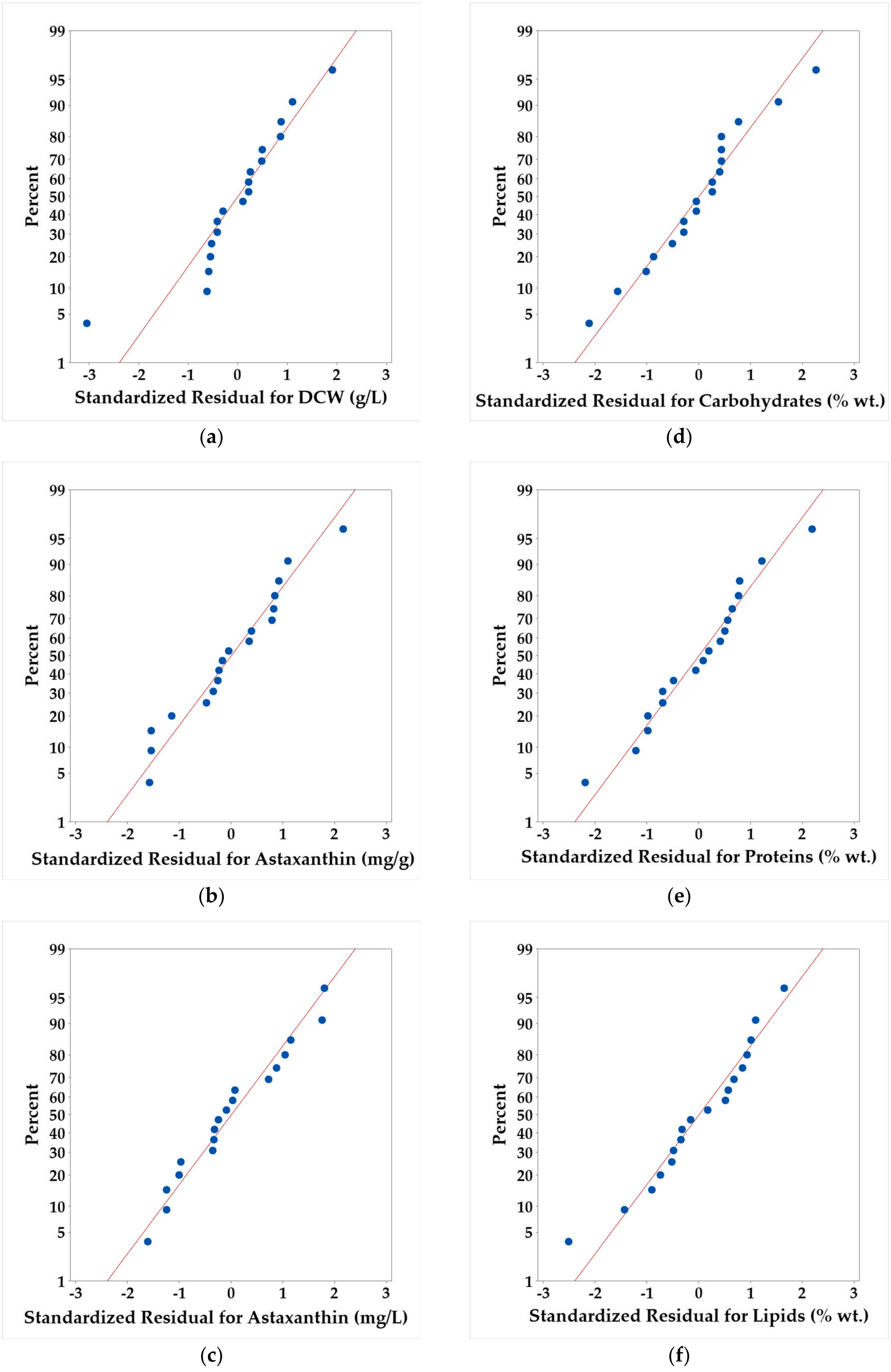

Figure S2. Normal probability plots of residuals for (a) Biomass; (b) Astaxanthin content; (c) Astaxanthin concentration; (d) Carbohydrate accumulation; (e) Protein accumulation and (f) Lipid accumulation. Models were fitted using 18 independent biological culture observations.

Table S1. Summary of residual diagnostics for the additive main-effects models fitted to the day-12 responses.

Response	AD statistics	*p*-value	Residuals-vs-fitted assessment	Flagged standardized residual	Overall assessment
Dry cell weight (g/L)	0.703	0.055		Observation 16: -3.05	Normality not rejected; flagged valid observation retained
Astaxanthin (mg/g)	0.343	0.450		Observation 14: +2.16	Normality not rejected
Astaxanthin (mg/L)	0.342	0.452	No evident systematic pattern or pronounced change in dispersion	None	Normality not rejected
Carbohydrates (% wt.)	0.351	0.430		Observation 4: +2.25 Observation 7: -2.11	Normality not rejected
Proteins (% wt.)	0.246	0.718		Observation 5: -2.19; Observation 14: +2.18	Normality not rejected
Lipids (% wt.)	0.297	0.552		Observation 18: -2.51	Normality not rejected

AD denotes the Anderson-Darling statistic, and standardized residuals are reported for observations flagged by Minitab as having comparatively large residuals. At $\alpha = 0.05$, residual normality was not rejected for any fitted response. Flagged observations were retained because a large residual does not, by itself, demonstrate measurement error or justify exclusion.

3. Model validation

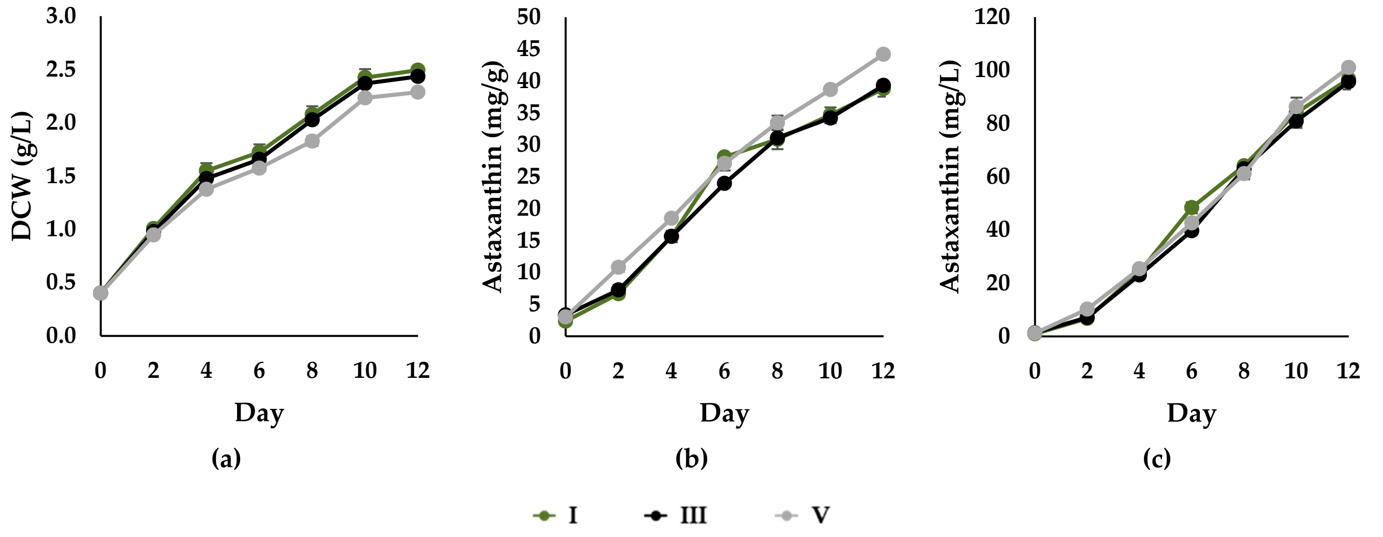

Figure S3. Production of (a) Total Biomass; (b) Astaxanthin content and (c) Astaxanthin concentration. I: Culture I – main experiment; III: Culture I – repeated experiment and V: optimal culture – experimental response. Error bars represent the standard deviation of the two biological replicates.
